# Dendritic fibromyxolipoma with intramuscular involvement: A case mimicking slow flow vascular malformation on imaging

**DOI:** 10.1016/j.radcr.2023.12.035

**Published:** 2024-01-13

**Authors:** Hashim AlSalman, Hassan alsayegh, Nada Elmukhtar, Ahmad AlZahrani, Salman AlBakheet, Qasem AlAlwan, Ahmed Almuslim, Haniyya AlRehaily, Mohammed Al Salman

**Affiliations:** a Department of Medical Imaging, King Abdulaziz Medical City, Riyadh, 11426, Saudi Arabia; b Department of Medical Imaging, King Fahad Hospital, Hofuf, 36441, Saudi Arabia; c Department of Pathology, King Fahad Hospital, Hofuf, 36441, Saudi Arabia; d Department of Medical Imaging, AlMoosa Specialist Hospital, al-Mubarraz, 36441, Saudi Arabia

**Keywords:** Dendritic fibromyxolipoma, Myxoid tumor, MRI

## Abstract

Dendritic fibromyxolipoma (DFML) is a benign, very rare, and slow-growing soft tissue tumor commonly involving the muscular fascia of the foot, calf, shoulders, back, or head and neck muscles. Many authors consider dendritic fibromyxolipoma a variant of spindle cell lipoma composed of a plexiform vascular pattern, dendritic cytoplasmic processes, and keloidal collagen. Only a few cases have been reported in the shoulder region, and the presented case represents the second case in English literature whose histopathology showed intramuscular involvement. Recognition of such an entity is essential because it is considered a scarce type of benign tumor that can be mistaken for other aggressive neoplasms of myxoid pathology. We present a case of a dendritic fibromyxolipoma around the right shoulder with intramuscular involvement to the superficial fibers of the right trapezius muscle.

## Introduction

Dendritic fibromyxolipoma (DFML) is considered a very rare, distinct, benign, superficial, and slow-growing soft tissue tumor first described in 1998 by Suster et al. [1]. These soft tissue tumors usually have a mixture of several mature fat cells and small spindles to stellate cells, sharing very morphologically similar features to other mesenchymal tumors of myxoid components, like myxoid liposarcoma and solitary fibrous tumor [2]. They commonly involve superficial fascia and may occasionally be seen in muscles. DFML also has histological features similar to spindle cell lipomas, usually characterized by an abundant myxoid component and stellate cells with a dendritic process [3]. Less than 70 cases were described in the literature. Here we present a case of biopsy-proven right upper back/right shoulder DFML, mistaken to be a slow flow vascular malformation on imaging.

## Case report

A 38-year-old male presented to primary health care complaining of a painless growing swelling around the right upper back/right shoulder. The swelling was incidentally felt and painless during rest, with mild aggravated pain during flexion and extension of the shoulder joint that did not affect the quality of life. No numbness or paresthesia was reported. On examination, the swelling was firm, mobile, and nontender, with no skin changes.

## Imaging

**Fig. 1:**
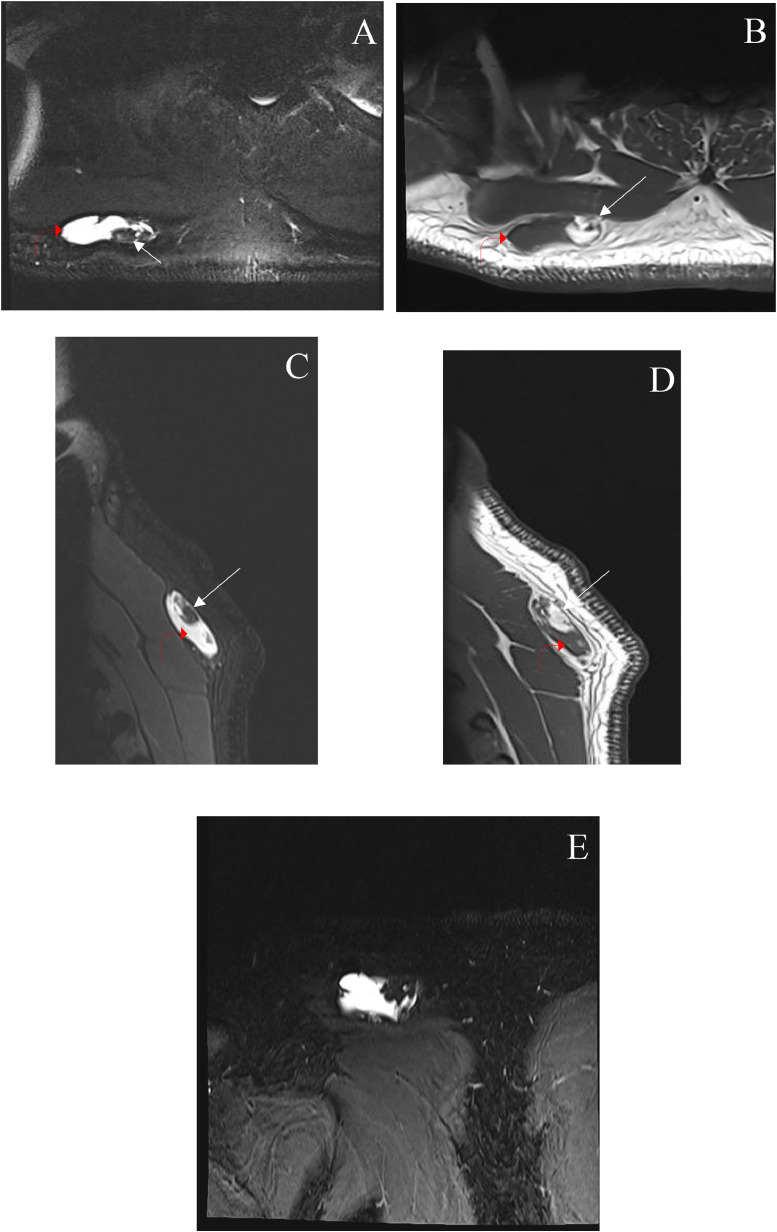
Fat-saturated axial T2 weighted image (A) axial T1 weighted image (B) at the level of the right shoulder/upper back shows an oval-shaped lobulated subcutaneous lesion of predominantly T2 hyperintensity (bent red arrows). T1 weighted image shows T1 hypointensity with fatty components (arrows). Fat-saturated sagittal T2 weighted image (C) sagittal T1 weighted image (D) and coronal T2 weighted image with fat saturation and (E) show the corresponding mass superficial to the right trapezius muscle.

**Fig. 2:**
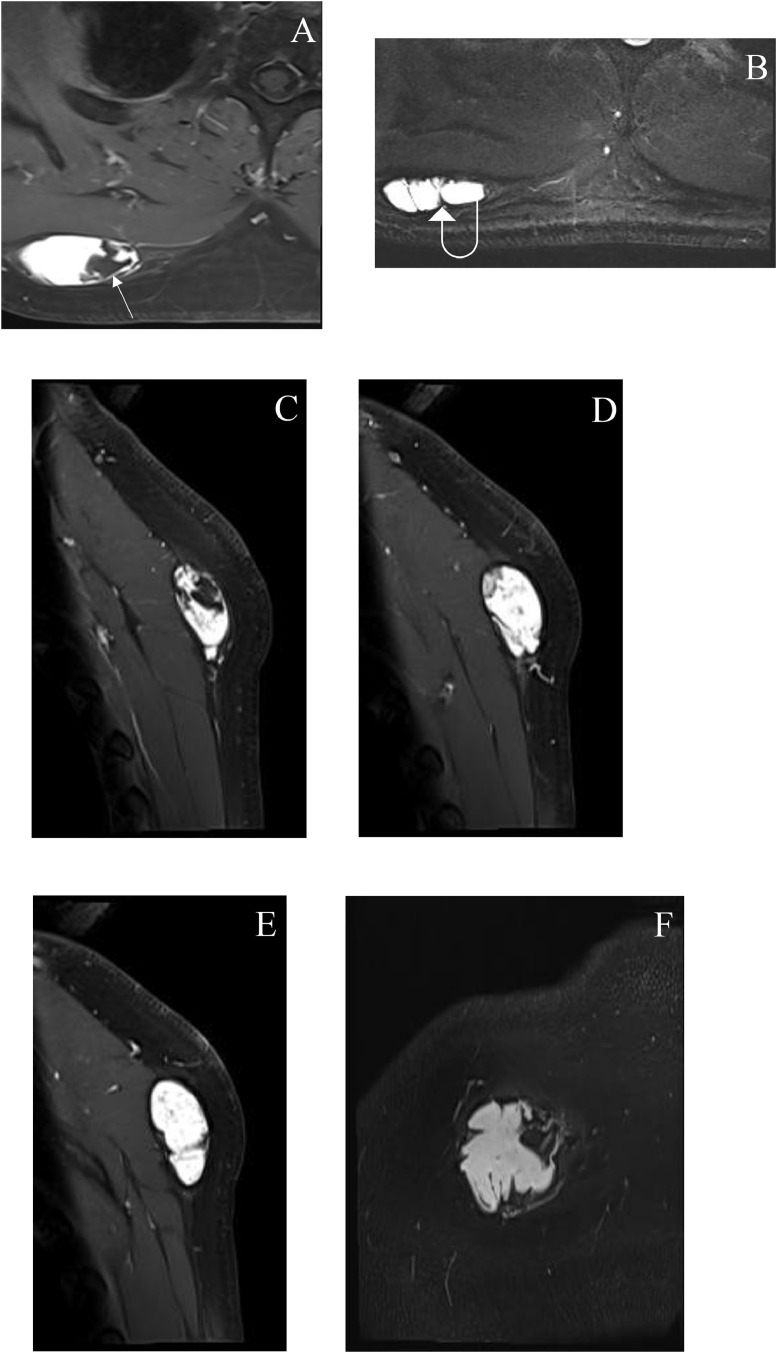
Postcontrast axial T1 weighted images at different levels (A and B) sagittal (C, D, and E) and coronal (F) show avid enhancement of the lesion and no enhancement of the fatty (arrow) and septal (curved arrow) components. No definite invasion into the surrounding structures.

Routine noncontrast right shoulder magnetic resonance imaging (MRI) was done, which revealed a lobulated subcutaneous lesion and fatty components (Fig. 1). The soft tissue component was predominantly hyperintense on T2-weighted images and predominantly hypointense on T1-weighted images. There were no local aggressive imaging features (Fig. 1). The mass was thought to be related to slow-flow vascular malformation like a hemangioma. However, based on the first MRI's findings, further evaluation with contrast-enhanced MRI and vascular surgery consultation was recommended. A postgadolinium MRI was done 3 months later, which revealed avidly enhancing soft tissue mass. Because of its characteristic appearance and avid enhancement, the mass was considered a slow-flow vascular malformation (hemangioma) (Fig. 2).

## Surgical pathology

**Fig. 3:**
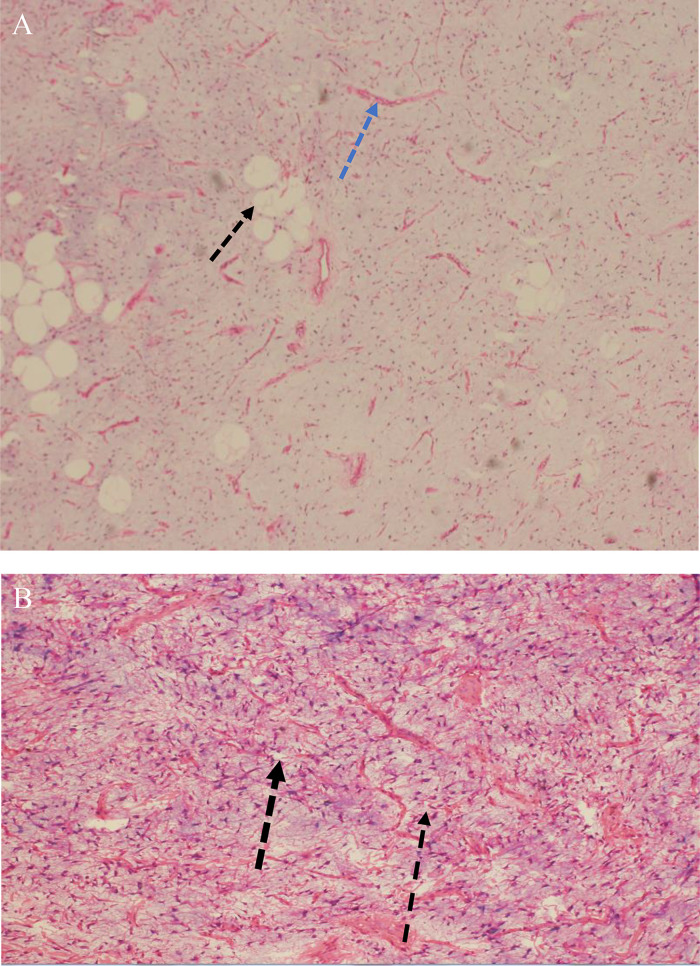
(A) Microscopic examination of hematoxylin and eosin (H&E) (low-power view 20x), revealing mature adipocytes (black arrow) with admixed spindle cells in loose connective tissue stroma containing thin walled blood vessels (blue arrow) and (B) Hematoxylin and eosin (high-power view 40X) demonstrated a proliferation of spindle-stellate cells (black arrow) embedded in the loose myxomatous stroma.

**Fig. 4:**
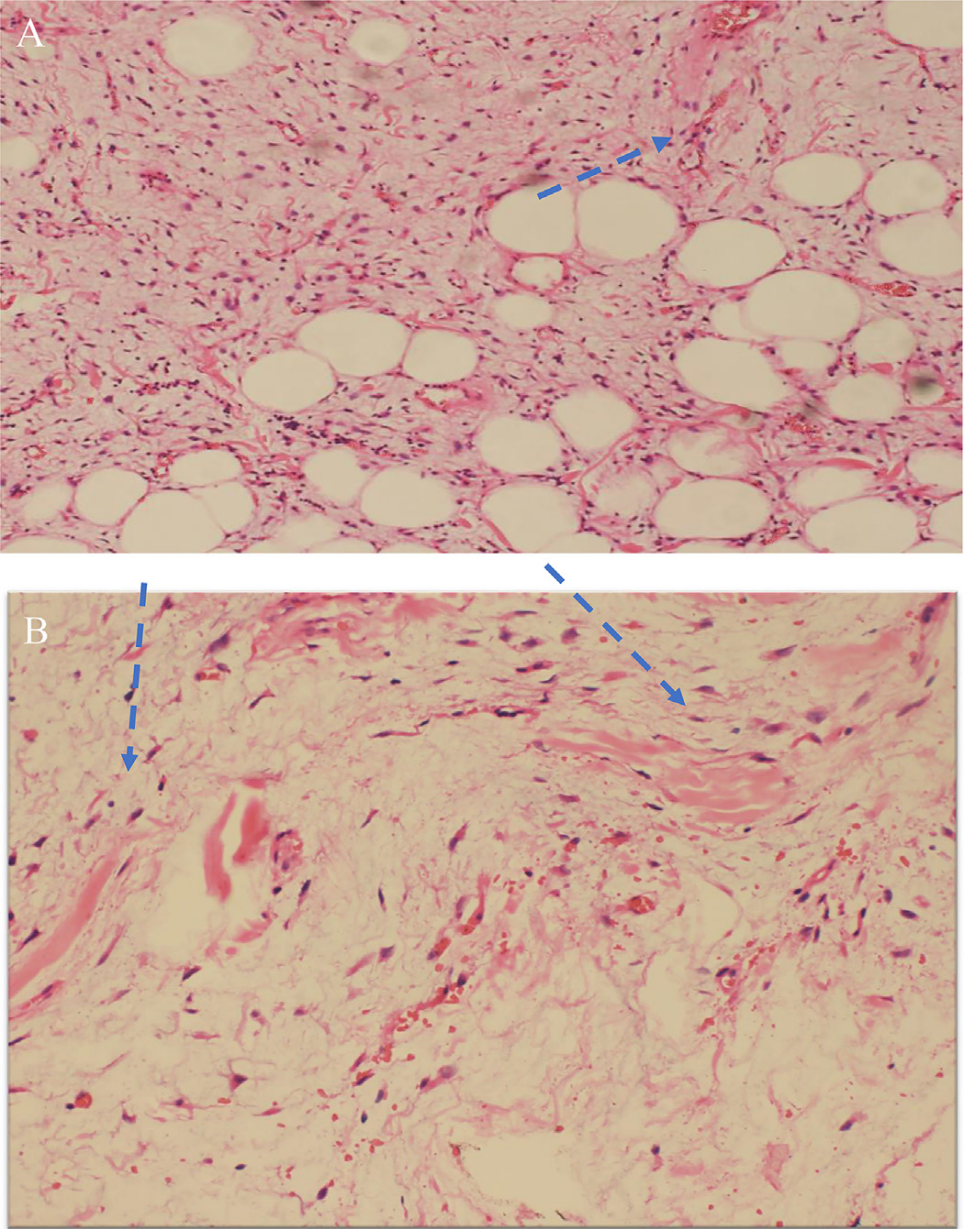
(A) Microscopic examination of H&E (40X shows infiltration of skeletal muscle fibres by proliferating stellate cells.(blue arrow) and (B) Splaying of skeletal muscle fibres(blue arrow heads) by stellate cells.

**Fig. 5:**
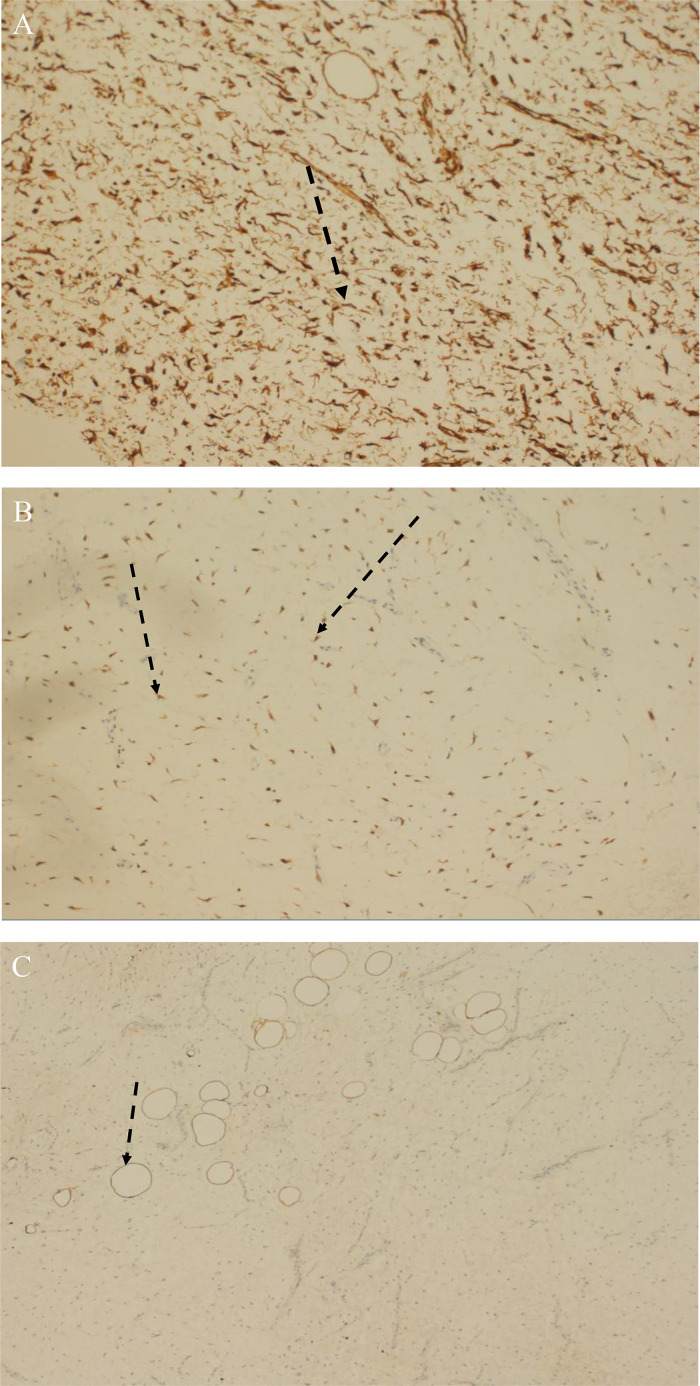
(A) Shows CD34 immunoreactive stellate cells (black arrow), (B) Shows Bcl-2 positive stellate cells (black arrows) and (C) highlights S100 positive adipocytes (black arrow), negative staining in stellate cells.

The mass was removed by the vascular surgeon during day surgery with a wide local excision, no frozen section, and no neoadjuvant treatment. The patient left the hospital on the same day in good condition without postoperative complications and was scheduled for a follow-up with the vascular surgery clinic after 2 months. The gross examination of the surgically excised mass demonstrated soft tan light grey mass, measuring 4 × 3.5 × 2 cm with soft gelatinous cut surface. The histopathological examination reveals an adipocytic neoplasm comprising of mature adipocytes with admixed proliferation of variably cellular spindled-stellate cells having delicate cytoplasmic processes with interspersed ropy collagen fibres embedded in myxomatous rich, well vascularized stroma (Fig. 3). There is infiltration with splaying of skeletal muscle fibres by proliferating stellate cells along with the paucicellular myxomatous rich stroma (Fig. 4). Immunohistochemical stains with adequate control were performed & showed CD34& Bcl-2 immunoreactive stellate dendritic cells with diffuse strong pattern of staining (Fig. 5) &S100 negative stellate cells (Fig. 5C) .The diagnosis of dendritic fibromyxolipoma was made.

## Clinical follow-up

The patient was doing well after a 2-month follow-up at the vascular surgery outpatient clinic, with no eventful presentation upon his initial visits. One year later, the patient started complaining of minimal pain at the site of surgery and revisited the vascular surgeon. On examination, no swelling was appreciated. The patient only complained of minimal pain, which was expected at the site of surgical scars, so the patient was given analgesia as needed.

## Discussion

We present a case of a histopathologically proven DFML involving the right upper back/right shoulder with intramuscular involvement, initially thought to be a type of slow-flow vascular malformation by imaging. DFML is a rare entity defined as a benign, slow-growing tumor of the soft tissues with many clinical and pathological similarities to the rare, myxoid variant of spindle cell lipoma and solitary fibrous tumor with myxoid stroma [3], [4], [5], [6], [7], [8], [9]. Most of the literature reports considered this tumor a spindle cell lipoma variant with special characteristics [[3], [4], [5], [6], [7], [8], [9],[10], [11], [12], [13]]. The usual locations of involvement described in the literature are the shoulder, chest wall, head, and neck subcutis, groin, calf, foot, and back. Limbs, including toes, were less frequently involved [[3], [4], [11], [13]]. After reviewing the PubMed database and some Chinese literature, we only found 62 reported and cited DFML cases, which included 51 males and 11 females [10]. Less frequently, DFML cases were reported to involve the intramuscular tissue, forearm, lower-lip region, inguinal region, and perineum region [13]. Moreover, only a few cases in the English literature describe DFML of the back with intramuscular location [[7], [8],^11^]. DFML shows a wide age range from 24 to 81 years, with 52 male and 11 female cases, including the current reported case [10,11].

The most common clinical manifestation of DFML is a gradually growing mass without associated symptoms. The time taken by the patients to first notice the mass ranged from 1 month to 13 years [10]. When the involvement is superficial, the usual clinical presentation is a painless tumor mass for an extended period of time [10]. MRI is usually the best approach to evaluate the soft tissue mass further. DFML has an abundant amount of myxoid stroma with a large plexiform vascular pattern sharing similarity and creating confusion with more aggressive lesions such as myxoid liposarcoma. DFML may also share similar features with other aggressive lesions, including low‐grade fibromyxoid sarcoma, myxo-fibrosarcoma, and myxoid synovial sarcoma [3,4,13]. DFML MRIs reveal highly variable findings with usually well-circumscribed margins, mixed signals on the T1& T2-weighted images, and the presence of fatty components [[11], [13]]. DFML usually has nonspecific imaging features and clinical presentation, and its definite diagnosis is made through histopathological examination [10]. Still, diagnostic imaging techniques are not always able to confidently differentiate between benign and aggressive fatty tissue tumors like lipomas and liposarcomas. The distinction still relies on histopathology [9].

In the presented case, the mass imaging findings were almost similar to slow-flow vascular malformation, as no aggressive features were observed in the MRI images. Surprisingly, the histopathology assessment proved the DFML diagnosis, making this the first DFML case mimicking slow flow vascular malformation on imaging with histopathologically proven intramuscular involvement. Considering the imaging along with histopathology findings, the differential diagnosis for DFML includes soft tissue neoplasms containing myxoid spindle cells, which includes the myxoid variant of spindle cell lipoma, solitary fibrous tumor with myxoid stroma, myxoid liposarcoma, myxoid synovial sarcoma, fibromyxoma, myxoid perineuroma, and superficial angiomyxoma. Ultimately, the DFML diagnosis was reached based on the typical histopathological pattern. It is important for radiologists and pathologists to be aware of the characteristics of such a tumor. This will aid in a better diagnostic approach and avoid misdiagnosing, overestimating, or underestimating this tumor in contrast to other more aggressive soft tissue neoplasms.

## Conclusion

We presented an unusual case of DFML of the upper back/shoulder mimicking slow-flow vascular malformation with biopsy-proven intramuscular involvement. This case had an unusual imaging feature, size, and location. It is crucial to clearly identify such entity to avoid misdiagnosis and burden on the patient with wrong management. The diagnosis was made by tracing the typical histopathological criteria of DFML and excluding other features of malignancy.

## Patient consent

Informed written consent was obtained from the patients or patient relatives for publication of the case series and all imaging studies. Consent form on record.
